# Reverse engineering for reconstructing baseline features of dry age-related macular degeneration in optical coherence tomography

**DOI:** 10.1038/s41598-022-27140-8

**Published:** 2022-12-31

**Authors:** Shuxian Wang, Ziyuan Wang, Srimanasa Vejalla, Anushika Ganegoda, Muneeswar Gupta Nittala, SriniVas Reddy Sadda, Zhihong Jewel Hu

**Affiliations:** 1 Doheny Eye Institute, 150 North Orange Grove Boulevard, Room 251, Pasadena, CA 91103 USA; 2 University of North Carolina at Chapel Hill, Chapel Hill, NC 27514 USA

**Keywords:** Predictive markers, Image processing, Machine learning

## Abstract

Age-related macular degeneration (AMD) is the most widespread cause of blindness and the identification of baseline AMD features or biomarkers is critical for early intervention. Optical coherence tomography (OCT) imaging produces a 3D volume consisting of cross sections of retinal tissue while fundus fluorescence (FAF) imaging produces a 2D mapping of retina. FAF has been a good standard for assessing dry AMD late-stage geographic atrophy (GA) while OCT has been used for assessing early AMD biomarkers beyond as well. However, previous approaches in large extent defined AMD features subjectively based on clinicians’ observation. Deep learning—an objective artificial intelligence approach, may enable to discover ’true’ salient AMD features. We develop a novel reverse engineering approach which bases on the backbone of a fully convolutional neural network to objectively identify and visualize AMD early biomarkers in OCT from baseline exams before significant atrophy occurs. Utilizing manually annotated GA regions on FAF from a follow-up visit as ground truth, we segment GA regions and reconstruct early AMD features in baseline OCT volumes. In this preliminary exploration, compared with ground truth, we achieve baseline GA segmentation accuracy of 0.95 and overlapping ratio of 0.65. The reconstructions consistently highlight that large druse and druse clusters with or without mixed hyper-reflective focus lesion on baseline OCT cause the conversion of GA after 12 months. However, hyper-reflective focus lesions and subretinal drusenoid deposit lesions alone are not seen such conversion after 12 months. Further research with larger dataset would be needed to verify these findings.

## Introduction

Age-related macular degeneration (AMD) is a the most widespread cause of blindness in developed countries, progressing asymptomatically over several years^1^. Globally, around 200 million people are impacted by AMD and an estimated 9% of cases of blindness worldwide are caused by the disease^2^. While early-stage AMD is largely asymptomatic and intermediate-stage AMD is oligosymptomatic, late-stage AMD is highly symptomatic. AMD is characterized by decay in central vision that results in difficulty with tasks such as reading and identifying objects in early and intermediate stage and even severe vision loss or blindness in advanced stage. 28% of intermediate-stage AMD cases progress to advanced-stage within 5 years. Advanced-stage AMD is further subdivided into wet exudative (neovascular) and dry atrophic (nonneovascular) categories. Further, 19% of eyes with intermediate AMD develop geographic atrophy (GA) within 5 years. Transitioning or alternating between exudative and atrophic AMD is also possible^2^.

Diagnosis of AMD relies upon spectral-domain optical coherence tomography (SD-OCT)^3^ to determine changes in the macular region and, to a lesser degree, fundus autofluorescence (FAF) to differentiate between exudative and atrophic AMD^2^. OCT imaging produces a 3D volume consisting of cross sections of the tissue while FAF imaging produces a 2D mapping of metabolic fluorophores in the ocular fundus. Further, FAF imaging can be used to identify regions of dry AMD atrophy^1^ and been widely used as gold standard for GA analysis due to its high image contrast. Increasingly, features from OCT imaging are being identified as biomarkers for stage and type of AMD^4^ due to its 3D nature. However, previous approaches in large extent defined AMD biomarkers or features subjectively based on clinicians' observation.

Recently, there has been work towards applying deep learning methods to identify and visualize AMD baseline biomarkers^5–7^ and AMD late stage atrophy^8^ in SD-OCT and FAF images that can diagnose AMD. Deep learning—an objective artificial intelligence approach, has an advantage over traditional machine learning methods in that it learns directly from data so there is no need to manually designate features. Deep learning may enable to discover 'true' salient features that could not be determined clinically or that might be determined subjectively previously.

Prior work has examined the role deep learning can play in automatically identifying and visualizing AMD late-stage atrophy and early biomarkers in OCT volumes. In particular, previous work in our group has demonstrated that such deep learning methods are able to detect GA in late stage AMD^8^ and identify known baseline AMD biomarkers^6^. Further, our group has demonstrated that the combination of deconvolutional networks with a U-Net architecture^8,9^ can reveal features most salient for anticipating the progression of AMD^7^. A modified U-Net model was trained to predict GA progression from en-face projections of OCT volumes with ground truth labels provided by annotated FAF images from a follow-up visit. The en-face projections were constructed by first segmenting various retinal layers using a method developed in our group^10^. The CNN then produces a segmentation result for each en-face image, and takes the strongest output of the group for the final segmentation result. The modifications to U-Net were to reshape filters to be square rather than rectangular (in order to better fit the square data) and add a visualization block to each layer. Visualization was performed via deconvnets^11^. In particular, using the segmentation result, the visualization blocks use transposed convolutions to invert the operations of the forward pass through the network; as a result, salient input features from the en-face images can be recovered and visualized.

Another method to visualize discriminatory features for AMD and its late-stage atrophy in OCT images is called multiscale class activation mapping (MS-CAM)^12^. In this method, OCT volumes are preprocessed to reduce noise and flattened along the RPE layer to further reduce spatial variance. Individual B-scans are passed through a pre-trained VGG-16 network (further fine-tuned on the GA segmentation task) to generate multi-scale feature maps. The maps are combined using a rescaling module and an attentional fully connected layer. Finally, a fully connected random field algorithm extracts positive and negative seeds, and a random walk algorithm on the positive and negative seeds produces the final segmentation result. This method was reported to produce near state-of-art segmentation performance measured by Jaccard index on proprietary datasets.

While our prior work has attempted to analyze OCT volumes to determine AMD biomarkers towards dry atrophic AMD at a single point in time or to compute the likelihood of progression from early or intermediate to late AMD atrophy^7^ in projected OCT 2D maps. In this project, we propose a novel reverse engineering approach to objectively identify and visualize early biomarkers for dry AMD in 3D volumes instead of 2D maps from baseline exams before significant dry AMD atrophy occurs. In particular, based on the backbone of a fully convolutional neural network (CNN), we segment GA regions and reconstruct early AMD features via deconvolutional networks in baseline 3D SD-OCT volumes by utilizing manually annotated GA regions from a follow-up visit as ground truth. The novel reverse engineering-based reconstruction maps are also compared with that from MS-CAM.

## Methods

**Figure 1:**
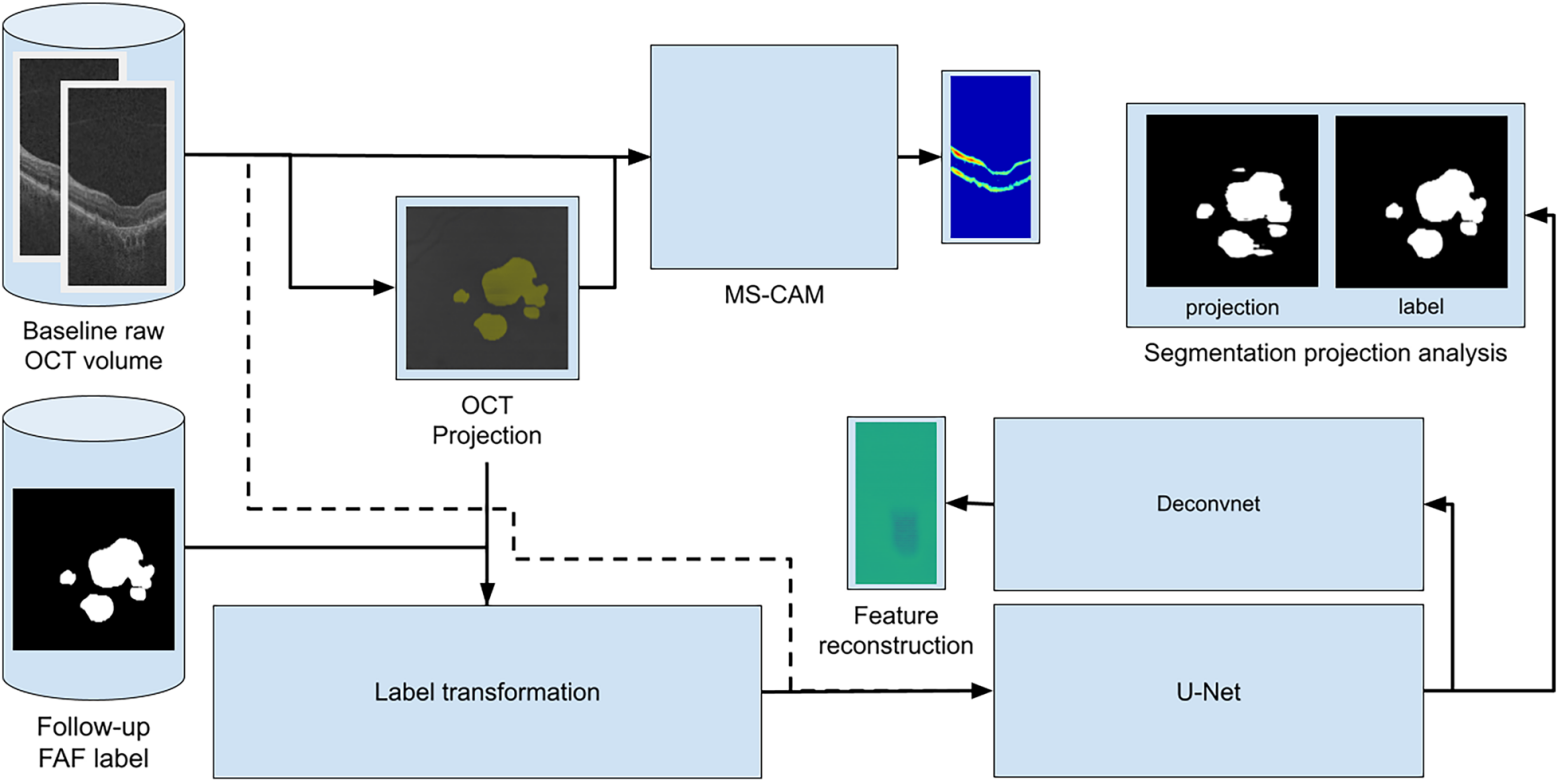
System overview.

We aim to reconstruct salient features in preliminary OCT exams that later develop GA to determine relevant biomarkers. We begin by gathering sets of baseline and follow-up FAF images such that the follow-up image indicates further progression of GA compared to baseline. We also include a baseline OCT volume for each set as the segmentation target. We generate labels for each B-scan of the OCT volumes by registering the follow-up FAF image with annotations to match the baseline FAF image, then projecting the registered annotation to align with individual B-scans. In this way, the dataset is designed to reverse-engineer the selection of biomarkers for GA in baseline images. We train the modified U-Net model to segment the projected annotation from B-scans. After training, we reconstruct salient features by using transposed convolutions to invert the convolutional layers of U-Net (see Fig. 1). We can generate statistics on our segmentation result and compare the salient features derived from our method with those obtained via MS-CAM. The reconstruction and MS-CAM results are illustrated in Figs. 4, 5, 6.

### Data

We use manual annotations from FAF images^13^ because it is currently the standard modality to quantify GA for AMD. Similarly, we use OCT as the baseline modality because of its 3D nature and prevalence in clinical use. Our dataset consists of 147 sets of baseline (patient first visit) spectral domain OCT (SD-OCT), baseline FAF, and follow-up (obtained at patient visit after 12 months) FAF images where the follow-up FAF image has manually annotated atrophy regions. Each set of images corresponds to a different patient. OCT images were recorded with a Cirrus HD-OCT camera (Carl Zeiss Meditec, Dublin, California) with image resolution 1024 × 512 × 128 centered on the fovea. FAF images were recorded with a Heidelberg confocal scanning laser ophthalmoscopy (Spectralis HRA + OCT, Heidelberg Engineering, Heidelberg, Germany) with image resolution varying from 496 × 596 to 1536 × 1536, all rescaled to 512 × 512. In order to make the OCT volumes and FAF images comparable, the OCT volumes are projected down the 1024 height dimension, then the 2D projections are rescaled to 512 × 512 before label transformation. All methods were carried out in accordance with relevant guidelines and regulations. For AMD eyes, ethics review and institutional review board approval from the University of California—Los Angeles were obtained. Informed consent from all subjects for the study participation and publication of identifying images were obtained.

### Label transformation

**Figure 2:**
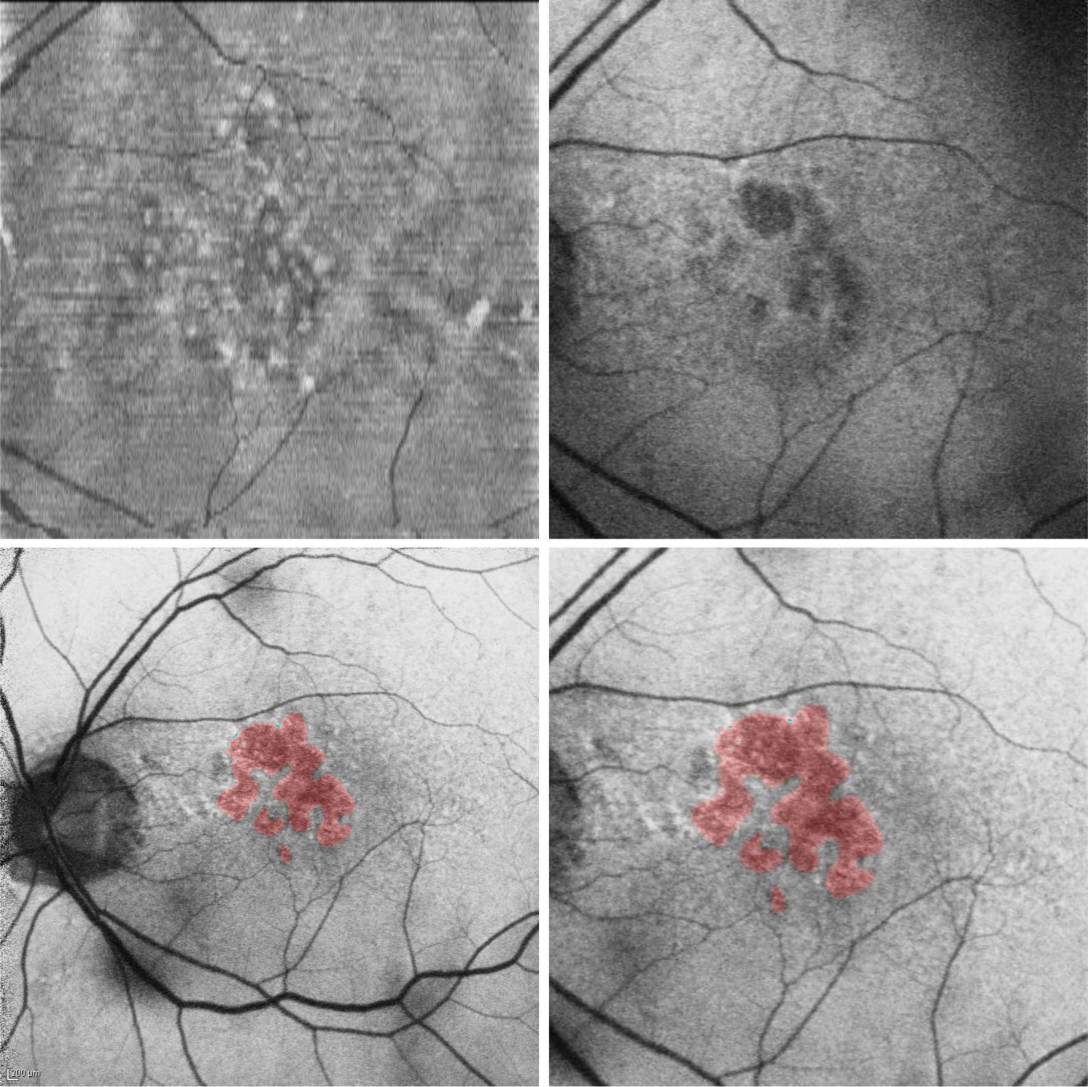
Example of registration with segmentation mask overlaid in red. Clockwise from top left: (1) projection of OCT volume of en-face B-scans, compressed along depth dimension (2) baseline FAF registered to match OCT projection (3) follow-up FAF after registering to match registered baseline FAF (4) follow-up FAF with segmentation mask.

Atrophy segmentation labels for OCT volumes were generated by registering the paired follow-up FAF images to the corresponding baseline volume projection. We manually selected keypoint correspondences in each pair to perform the registration. Keypoints were selected based on visual features in the images, such as significant blood vessels. By registering the entire FAF image, the segmentation annotation is also registered to match the anatomical features and position of the baseline FAF image. Thus, the geographic characteristics of the atrophy segmentation labels from the follow-up image are mapped to the baseline image. See Fig. 2 for an example of label transformation.

We project the 2D transformed atrophy segmentation down the height dimension of the OCT volume in order to obtain annotations for each B-scan. In particular, we resize the transformed segmentation and OCT projection to 512 × 128, then for each slice along the 128 depth dimension, project the width of the annotation down the 1024 height dimension. In order to better define the region of interest, we crop heights to be within the bounds of the ILM and C-S layers of the retina. The retina layers were automatically segmented using the method previously developed by our group^10^.

### Prediction of GA Progression from baseline OCT via reverse engineering

We build upon the previously-established good segmentation result provided by U-Net to predict atrophy progression from baseline OCT^8^ via reverse engineering. More specifically, we input the baseline OCT B-scan images to the deep CNNs of the U-Net for algorithm training. Note that the ground truth annotations are generated by the method described above and therefore reflect the GA growth since the baseline visit. Hence with inputting the baseline OCTs and the corresponding follow-up FAF label to the U-Net, the output of the network implies the follow-up GA growth from early features (i.e. early signs of morphological changes due to AMD) on baseline OCT.

We train the neural network using a Dice-based loss, which we define as

$$ L = \left( {1 - \frac{{2 \left| {y \cap \hat{y}} \right| + 1}}{{\left| y \right| + \left| {\hat{y}} \right| + 1}}} \right) + H\left( {y, \hat{y}} \right) $$

where $y$ is the predicted segmentation, $\widehat{y}$ is the ground truth annotation, and $H$ is the binary cross entropy function. We use the Adam optimizer with learning rate of 10^–4^. The input is B-scans and ground truth is given by the projected transformed atrophy segmentation labels. We used a Nvidia Quadro P5000 for training and testing.

We convert the output from the neural network to a binary classification. Recall that U-Net finishes with a softmax layer, which means that for each pixel location, we have an ordered tuple of positive and negative probabilities. Thus, we threshold the positive probability at 0.5 and take pixels above the threshold as a positive classification, and those below as a negative classification. This yields a binary mask over the image.

For each mask and its corresponding ground truth annotation, we compute a confusion matrix consisting of the true positive (TP), false positive (FP), true negative (TN), and false negative (FN) rates. We compute accuracy, sensitivity, specificity, and overlap as functions of the confusion matrix as defined as follows, then average over all segmentations to report the metrics listed in Table 1.

$$ accuracy = \frac{TP + TN}{{TP + TN + FP + FN}} $$

$$ sensitivity = \frac{TP}{{TP + FN}} $$

$$ specificity = \frac{TN}{{TN + FP}} $$

$$ overlap = TP $$

**Table 1:** Segmentation metrics for each fold of eightfold cross validation and aggregated result.

Fold	Accuracy	Sensitivity	Specificity	Overlap
0	0.95	0.61	0.97	0.67
1	0.96	0.62	0.98	0.71
2	0.96	0.51	0.98	0.58
3	0.96	0.50	0.97	0.54
4	0.93	0.65	0.93	0.66
5	0.93	0.56	0.94	0.59
6	0.95	0.62	0.97	0.63
7	0.93	0.73	0.94	0.77
Combined	0.95	0.60	0.96	0.65

### Feature reconstruction via reverse learning

**Figure 3:**
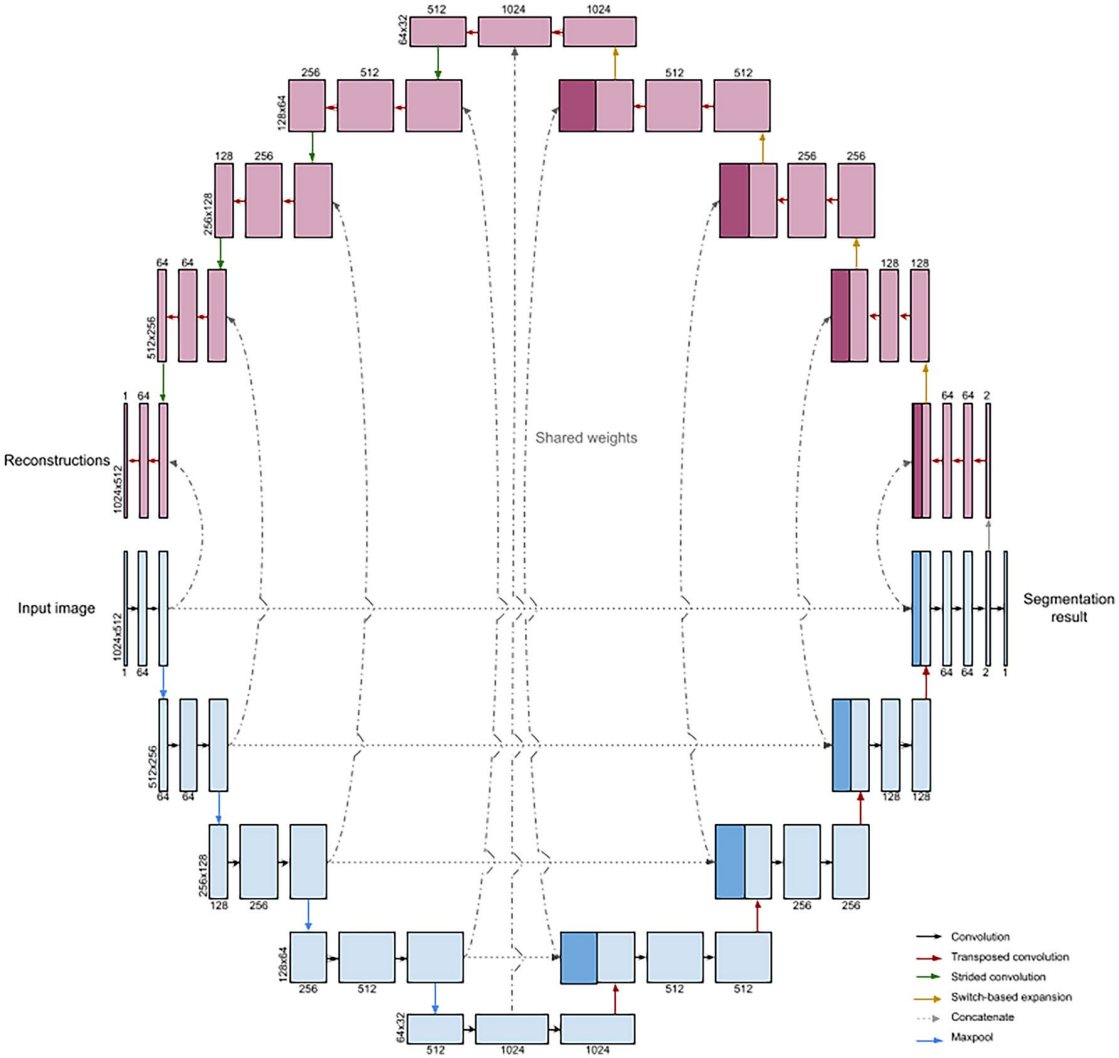
Architecture diagram for augmented U-Net.

We modify U-Net such that each convolution block is paired with a reconstruction block where a transposed convolution replaces the standard convolution but operations share weights following the method^11^ (see Fig. 3). The visualization block also operates in reverse, so that input to the visualization block is first passed through the activation before the transposed convolution. For max pooling layers, the forward operation stores the locations of the maximal values kept after pooling (switches in the terminology of Zeiler et al.) so that the inverse operation can place values back in the stored location and zero out remaining values. In this way, the visualization blocks together form a deconvnet that requires no additional training while producing reconstructions for each layer of the network. Through recovering the inputs to each block in this way, we produce reconstructions of the notable signals input into each layer of the forward pass. Thus, we can recover the significant features in the input B-scan from the segmentation result by iteratively reconstructing the inputs to each layer of the model. We examine the reconstruction results below.

### Class activation mapping

We compare our result with the feature map obtained via MS-CAM^12^, as described above. Since the target of MS-CAM is to extract significant features from B-scans just as we reconstruct significant features in input B-scans, we expect that the MS-CAM results will demarcate similar features as our reconstructions. We use the model trained by the authors for this task in order to better reproduce their result. We added postprocessing via Otsu thresholding with three classes, setting values below the higher threshold to zero, to reduce noise. Values above the threshold are retained without modification.

## Results

The key property of our model is its image representation. Image representation manifests as both segmentation accuracy in the network output and meaningful feature selection in the reconstructed input. Since we cannot quantitatively measure the reconstructed input features, we analyze them qualitatively in the Discussion section. Here, we focus on the segmentation performance instead.

We compare the OCT B-scan segmentation output to registered FAF manual ground-truth by averaging the OCT B-scan segmentation results over the depth dimension of the OCT volume and resizing the resulting OCT projection to square 512 × 512. We binarize this projected OCT segmentation result by computing the Otsu threshold for 2 or 3 classes and taking the threshold that gives the highest Dice score. With the optional second threshold, we can ignore more noise in particularly ambiguous intermediate results. We can then compute final metrics on the projected OCT segmentation and registered FAF ground truth for the evaluation of the automated segmentation results on OCT. Table 1 shows the accuracy, sensitivity, specificity, and overlap scores of the automated OCT segmentation against the registered FAF ground truth. We find an overall segmentation accuracy of 0.95, demonstrating the effectiveness of the image representation the U-Net has learned for this task.

## Discussion

**Figure 4:**
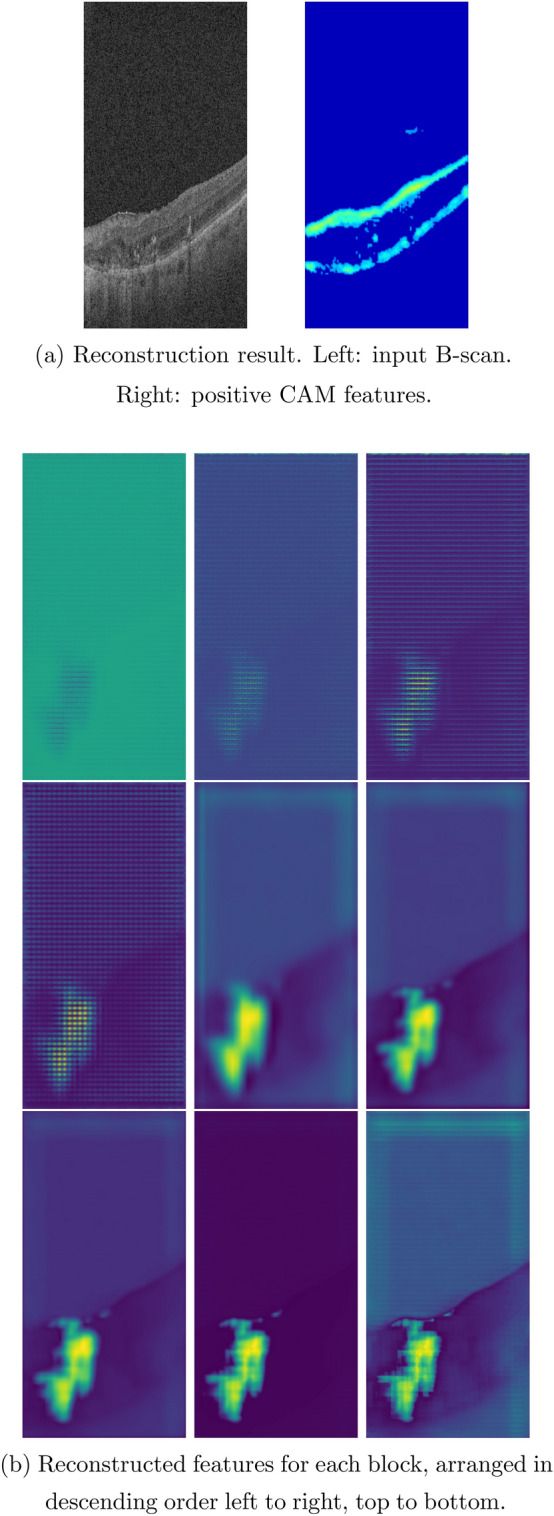
(**a**) Reconstruction result. Left: input B-scan. Right: positive CAM features. (**b**) Reconstructed features for each block, arranged in descending order left to right, top to bottom.

**Figure 5:**
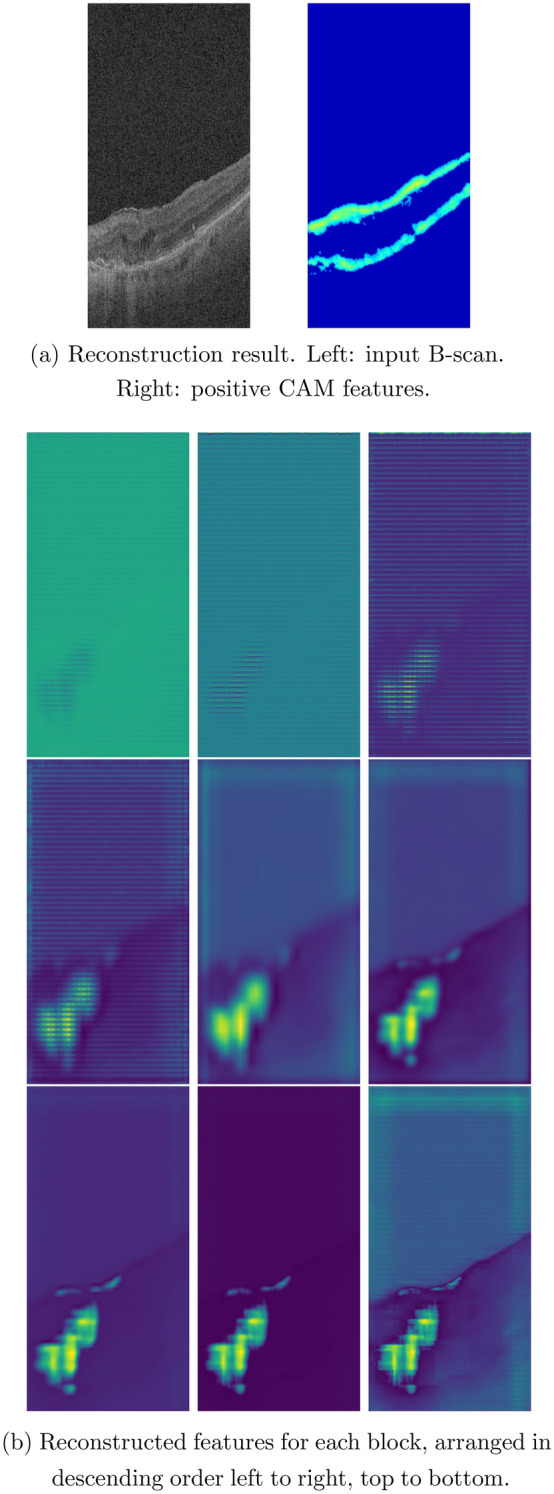
(**a**) Reconstruction result. Left: input B-scan. Right: positive CAM features. (**b**) Reconstructed features for each block, arranged in descending order left to right, top to bottom.

**Figure 6:**
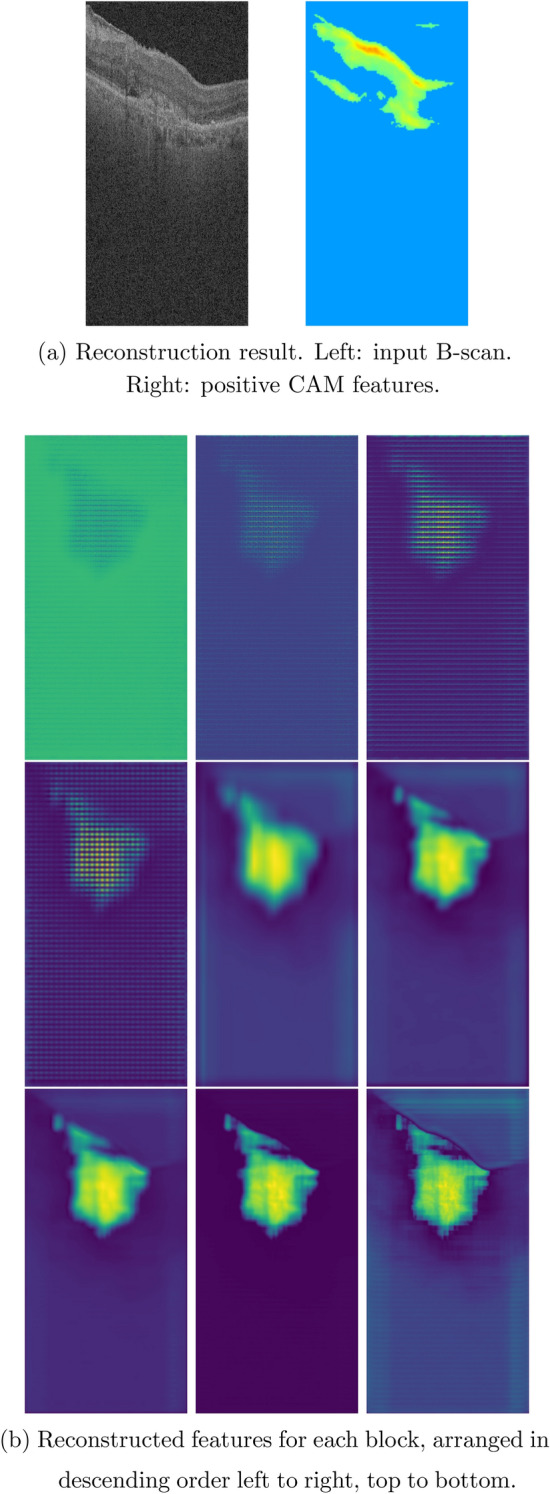
(**a**) Reconstruction result. Left: input B-scan. Right: positive CAM features. (**b**) Reconstructed features for each block, arranged in descending order left to right, top to bottom.

The reverse engineering scheme permits the identification and visualization of baseline salient input OCT features or early AMD biomarkers which result in the conversion to GA in the follow-up visit after 12 months. In this paper, we apply two visualization approaches of the feature reconstruction and the MS-CAM. Figures 4, 5, and 6 show three sets of examples of the reconstruction and MS-CAM results from three SD-OCT B-scans. From Fig. 4, one can see that the reconstruction results highlight an early AMD feature—a larger deteriorated (or collapsed) druse cluster region which mixes with a few hyper-reflective foci as shown on the corresponding baseline OCT B-scan and this region converts to GA after 12 months. There are a few relatively large individual hyper-reflective foci in the right side on the OCT B-scan. However, in the reconstructions, these individual hyper-reflective foci are not shown and they also do not convert to GA at month 12 visit. For the MS-CAM result, the activation map highlights the inner and outer retinal layers, and the hyper-reflective foci in the right side. However, the collapsed larger druse cluster area is not highlighted. Figure 5 visualizes the reconstructions and MS-CAM results from a B-scan of the same OCT volume as Fig. 4. Similarly, the reconstructions highlight a druse cluster (also mixing with a bit of tiny hyper-reflective foci) in the left side of the baseline OCT B-scan converting into GA in a follow-up visit at 12 months. The OCT B-scan includes several individual hyper-reflective focus lesions which are not shown in the reconstructions and these hyper-reflective lesions also do not progress to GA after 12 months. As a summary of the findings from Figs. 4 and 5, the reconstructions consistently highlight the druse clusters mixing with hyper-reflective foci on baseline OCT B-scans which result in GA in follow-up visit after 12 months. However, the MS-CAM maps do not show early AMD features consistently. The individual hyper-reflective foci are not shown in the reconstruction results and these lesions do not actually convert to GA in the follow-up visit at 12 months. It may imply that for the individual hyper-reflective foci, it may not convert to GA or may take longer time more than 12 months to convert to GA.

Additionally, Fig. 6 visualizes the reconstructions and MS-CAM results from a B-scan of a different OCT volume as Figs. 4 and 5. Similarly, the reconstructed results highlight a larger druse cluster area (mixing with a bit of tiny hyper-reflective foci) which progresses to GA in the follow-up visit after 12 months on the corresponding baseline OCT B-scan. There is subretinal drusenoid deposit (SSD) presented in the right side on the OCT B-scan. However, it is not showed in the reconstructions and after 12 months, this region does not show atrophy. For the MS-CAM result, the activation map highlights the inner and outer retinal layers, but does not highlight the larger druse cluster. Note that the B-scan in Fig. 4 and the B-scan in Fig. 6 are from different AMD subjects. However, the findings for individual hyper-reflective foci are the same. It may verify that for the hyper-reflective focus lesions, it may take longer time to convert to GA or may not convert to GA in the future.

Overall, the reconstructions consistently demonstrate baseline AMD features (or biomarkers) which result in GA development after 12 months. In our data, there are three types of AMD lesions (drusen, hyper-reflective foci, and SSD lesions) presented on baseline OCT which may convert to GA after the development of a period based on the reported literature. Among them, drusen mixing with hyper-reflective foci convert to GA in the follow-up visit at 12 months but SSD lesions and hyper-reflective foci are not seen such conversion and the reconstructions also do not highlight the area of those lesions.

## Conclusion

In this project, we develop a novel reverse engineering approach to objectively identify and visualize dry AMD features or early biomarkers on 3D OCT volumes from baseline exams before significant GA occurs. The approach bases on the backbone of a fully convolutional NN of the U-Net to predict GA regions and reconstruct early AMD features in baseline 3D OCT volumes by utilizing manually annotated GA regions from FAF from a follow-up visit as ground truth. The inputs of the U-Net are the baseline OCTs and the corresponding follow-up FAF labels, so the output of the network implies the follow-up GA growth from early AMD features on baseline OCT. In this preliminary exploration, compared with the manual ground truth, we achieve the baseline GA segmentation accuracy of 0.95 and overlapping ratio of 0.65.

Our approach has some limitations. (1) We are able to achieve high accuracy and specificity, but lower sensitivity and overlap ratio when computing metrics on the projected segmentation result compared to ground truth. This suggests that future work may focus on improving the segmentation result, which in turn may improve the reconstructed visualization of salient input features. (2) AMD overall develops to GA slowly, which may take several years to decades. In our data with relatively shorter follow-up period, individual hyper-reflective foci and SSD are not seen converting to GA. Whether these lesions would convert to GA or not, and whether these features can be correctly reconstructed would need longer longitudinal data and further research to verify.

## Data Availability

The image data utilized in this study are not publicly available due to the patients’ privacy and the violation of informed consent. However, they can be available from the corresponding author on reasonable request and official agreement.
